# Antiretroviral Strategies to Prevent Mother-to-Child Transmission of HIV: Striking a Balance between Efficacy, Feasibility, and Resistance

**DOI:** 10.1371/journal.pmed.1000169

**Published:** 2009-10-27

**Authors:** Dara A. Lehman, Grace C. John-Stewart, Julie Overbaugh

**Affiliations:** 1 Division of Human Biology, Fred Hutchinson Cancer Research Center, Seattle, Washington, United States of America; 2 Department of Medicine, University of Washington, Seattle, Washington, United States of America; 3 Department of Global Health, University of Washington, Seattle, Washington, United States of America; 4 Department of Epidemiology, University of Washington, Seattle, Washington, United States of America

## Abstract

Dara Lehman and colleagues discuss a randomized trial that found that adding up to a week of twice-daily zidovudine+lamivudine to single-dose nevirapine reduces the risk of resistance in mothers and infants.

Prevention of mother-to-child transmission (MTCT) of HIV has been both a great success and a continued challenge. Today, in resource-rich countries, new infant infections are a rare event. However, nearly 400,000 infant HIV-1 infections still occur each year in settings in which highly active antiretroviral therapy (HAART), elective caesarean sections, and safe alternatives to breastfeeding are not readily available. In these settings, various short-course antiretroviral therapies that include a single-dose of nevirapine (sdNVP) are used to prevent transmission to infants [1]. The sdNVP regimen effectively reduces MTCT by close to 50% and is an inexpensive and simple regimen, feasible for use in resource-limited settings [2],[3]. However, resistance to sdNVP arises commonly and quickly and can adversely affect the future treatment of NVP-exposed women [4]–[6]. Ideal alternatives to the sdNVP regimen would reduce the emergence of resistance while preserving efficacy and feasibility.

## Alternatives to a Single Dose of NVP to Reduce MTCT

A variety of alternative regimens have efficacy in preventing MTCT in resource-poor settings. These include daily zidovudine (AZT) or zidovudine/lamivudine (AZT/3TC) combinations, short-course HAART, and infant-only prophylaxis [7]. When considering which strategy is most appropriate for use in resource-limited settings, data on transmission rates must be balanced with factors such as resistance, safety, feasibility, and adherence.

Despite the fact that it has been 10 years since sdNVP was shown to be efficacious, only about 10%–30% of pregnant women who need sdNVP in resource-poor settings have access to this affordable and simple regimen [3],[8]. This fact alone suggests that more complex regimens, including short-course HAART, may not be as rapidly scaleable for preventing MTCT, despite their superiority to sdNVP in preventing transmission and resistance [9],[10]. The approach of infant-only treatment, while avoiding resistance in the mother [11], is unable to prevent the transmissions that occur in utero or intrapartum, and does not avoid resistance in infants that do become infected. Thus, there is a compelling need for a regimen that approaches the simplicity of sdNVP while minimizing resistance.

## A Postpartum “Tail” of Antiretrovirals Reduces Resistance Following sdNVP

As described in a paper in this issue of *PLoS Medicine*, Martinson et al. conducted a randomized trial to determine whether adding up to a week of twice-daily AZT/3TC to sdNVP would reduce the risk of resistance in mothers and infants [12]. The addition of AZT and 3TC (half-lives 1–2 h and 5–7 h, respectively) decreases the amount of time that NVP (half-life 45 h) would be present alone, potentially limiting selection pressure for resistance to emerge. At 6 wk postpartum, drug resistance in both mothers and infants was reduced by over 80% in the sdNVP plus AZT/3TC arms, compared to sdNVP alone. In addition, at 2 wk postpartum, viral loads were lower in the women on combination treatment compared to those who received sdNVP alone, which may also have contributed to the observed reduction in resistance. Not only did the overall percentage of women and children with resistance decrease, but following sdNVP plus the “tail” of AZT/3TC, fewer acquired multiple mutations and resistance appeared to fade more quickly.

These data have already had a global impact. Preliminary analysis of the data from this research study was presented at the Conference on HIV Pathogenesis and Treatment in 2005, and motivated a change in the World Health Organization (WHO) recommendations for preventing MTCT in resource-limited settings [1],[13]. Before these results were available, sdNVP alone was the standard treatment recommended by the WHO, despite the well-known risk of resistance. It was simply the most feasible regimen at the time. Today, the current WHO recommended regimen (AZT antepartum, sdNVP plus AZT/3TC intrapartum, followed by AZT/3TC for 7 d postpartum) is based on the results presented by Martinson, et al. as well as a subsequent study that showed similar findings [1],[14]. More recent data suggest that even simpler tail regimens may be possible [15].

Some important questions remain. The tail combination regimens provide a shorter duration of single drug selection pressure compared to sdNVP alone, and result in a reduced prevalence of drug resistance mutations that are detected by the population-sequencing assays used. However, the addition of a 1-wk tail may not be enough to completely eliminate NVP selection pressure, and it remains possible that resistant virus still arises, but only at low levels because of the more limited period of selection. Whether low-level resistance arises following these regimens, and whether it has clinical relevance, remains unclear and requires testing with more sensitive drug resistance assays [6].

Assuming that there is not a large amount of lurking resistance that is below the detection limit of the assays used in the study presented, the approach of sdNVP plus AZT/3TC to prevent MTCT may strike the right balance of a feasible regimen that minimizes resistance in settings where HAART remains to be implemented.

**Linked Research Article**

This Perspective discusses the following new study published in *PLoS Medicine*:

McIntyre JA, Hopley M, Moodley D, Eklund M, Gray GE, et al. (2009) Efficacy of Short-Course AZT Plus 3TC to Reduce Nevirapine Resistance in the Prevention of Mother-to-Child HIV Transmission: A Randomized Clinical Trial. PLoS Med 6(10): e1000172. doi:10.1371/journal.pmed.1000172

## Abbreviations

3TC: lamivudine
AZT: zidovudine
HAART: highly active antiretroviral therapy
MTCT: mother-to-child transmission
NVP: nevirapine
sdNVP: single-dose of nevirapine
